# Supplementary material for: Combined Multimorbidity and Polypharmacy Patterns in the Elderly: A Cross-Sectional Study in Primary Health Care
Source: Int J Environ Res Public Health. 2021 Sep 1;18(17):9216. doi: 10.3390/ijerph18179216 (PMC8430667; doi:10.3390/ijerph18179216)

Figure S1. Validation indices [31].

In total, our model contained a range of 4 to 8 clusters, as identified by the validation indices below. According to clinical relevance, it was decided that the best solution was composed of 7 clusters.

Fukuyama index average across increasing number of clusters; optimum index is minimum.

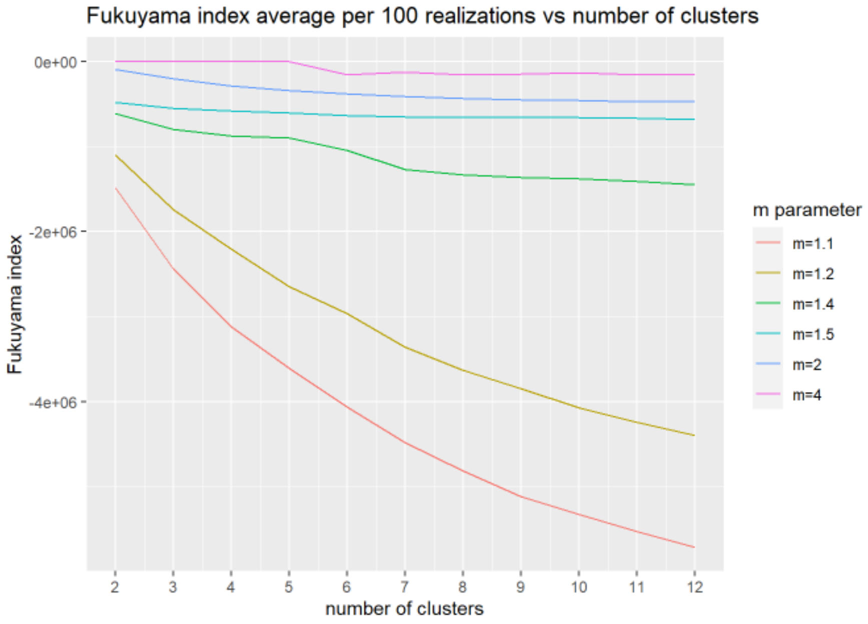

Xie-Beni index average across increasing number of clusters; optimum index is minimum.

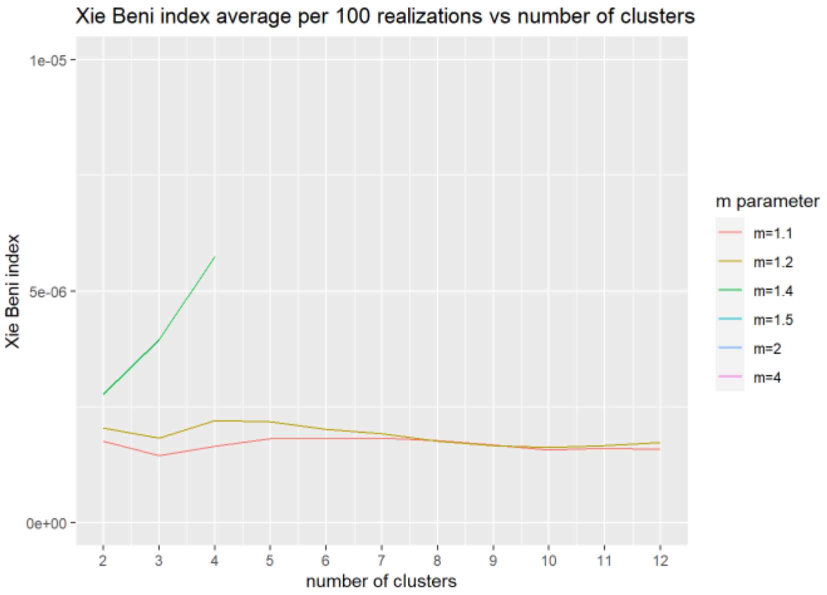

Partition coefficient index average across increasing number of clusters; optimum index is maximum.

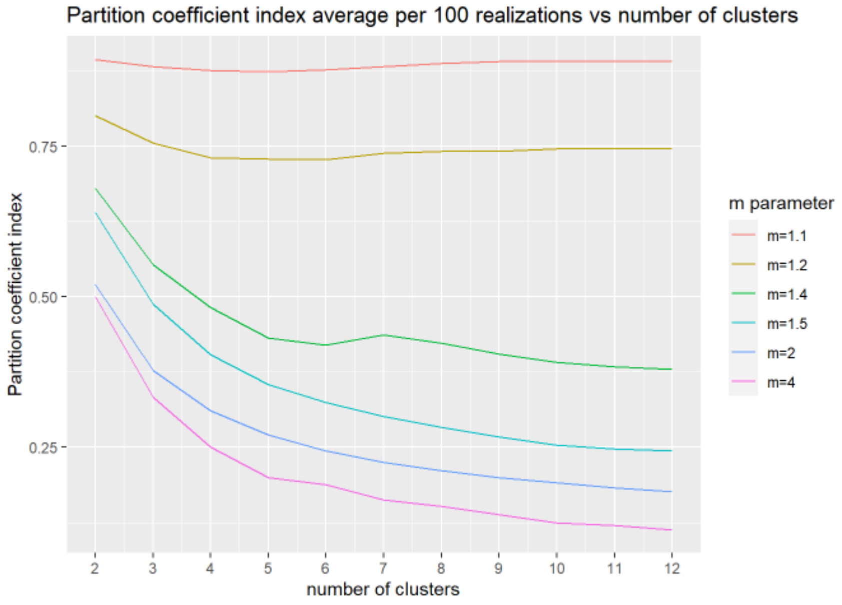

Partition entropy index average across increasing number of clusters; optimum index is minimum.

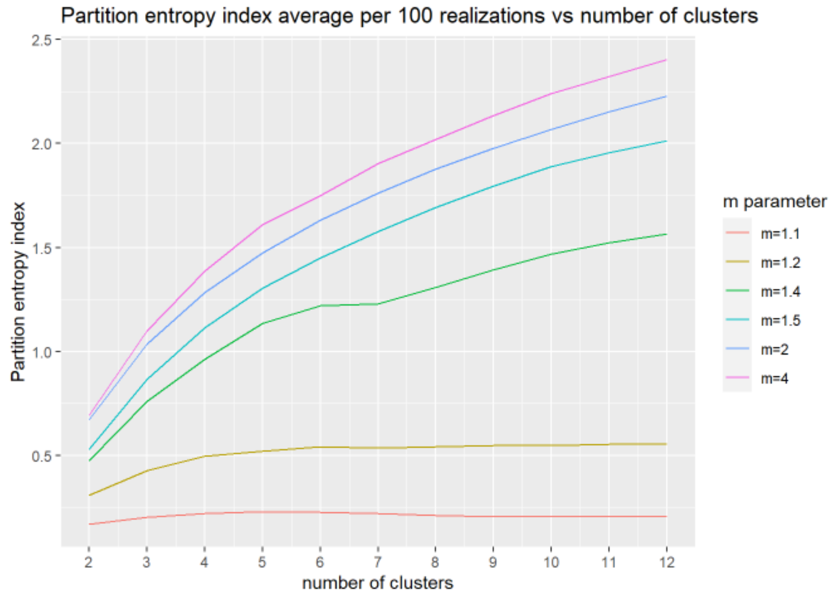

Calinski-Harabasz index average across increasing number of clusters; optimum index is maximum.

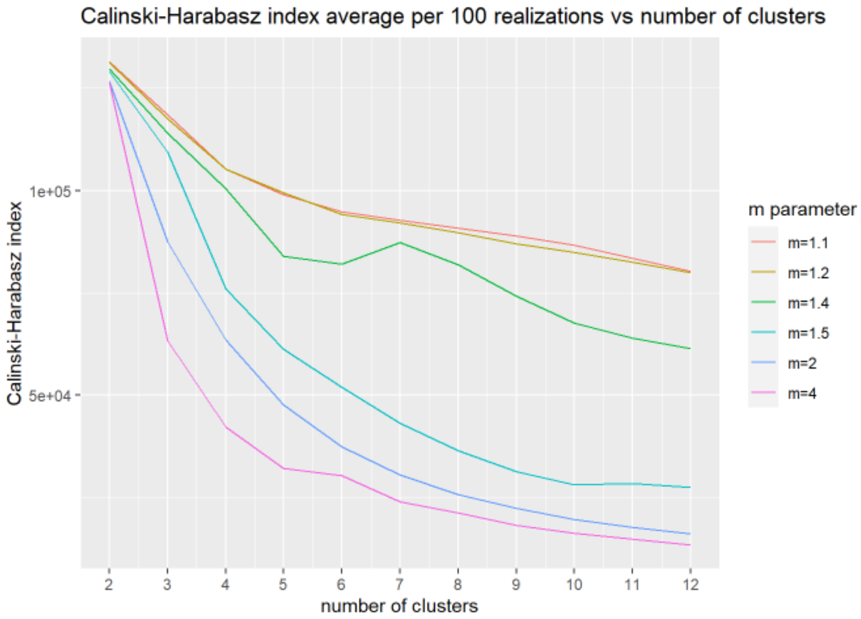

Supplement: Supplementary file 1 [file ijerph-18-09216-s001.zip › Supplementary_Figure S1.pdf]
