# Supplementary material for: Combined Multimorbidity and Polypharmacy Patterns in the Elderly: A Cross-Sectional Study in Primary Health Care
Source: Int J Environ Res Public Health. 2021 Sep 1;18(17):9216. doi: 10.3390/ijerph18179216 (PMC8430667; doi:10.3390/ijerph18179216)
Supplement: Supplementary file 1 [file ijerph-18-09216-s001.zip › Supplementary_Table S1.pdf]

Table S1. Mapping of SNAC-K chronic disease categories and their associated ATC drugs categories.

ALLERGY

ICD-10 codes and labels included in chronic disease category		*ATC-5 codes and labels for drugs associated to chronic disease category. Includes none or any of the following:*	
J301	Allergic rhinitis due to pollen	R01AD	Corticosteroids (nasal use)
J302	Other seasonal allergic rhinitis	R03AC	Selective beta-2-adrenoreceptor agonists
J303	Other allergic rhinitis	R03AK	Adrenergics in combination with corticosteroids or other drugs, excl. anticholinergics
J304	Allergic rhinitis, unspecified	R03BA	Glucocorticoids (inhalation)
J450	Predominantly allergic asthma	R03BB	Anticholinergics
K522	Allergic and dietetic gastroenteritis and colitis	R06AX	Other antihistamines for systemic use
L20	Atopic dermatitis	D07AC	Corticosteroids, potent (group III) (topical use)
L23	Allergic contact dermatitis		
L500	Allergic urticaria		
Z516	Desensitization to allergens		

ANEMIA

ICD-10 codes and labels included in chronic disease category		*ATC-5 codes and labels for drugs associated to chronic disease category. Includes none or any of the following:*	
D50	Iron deficiency anaemia	B03AA	Iron bivalent, oral preparations
D51	Vitamin B12 deficiency anaemia	B03AB	Iron trivalent, oral preparations
D52	Folate deficiency anaemia	B03BA	Vitamin B12 (cyanocobalamin and analogues)
D53	Other nutritional anaemias	H02AB	Glucocorticoids (systemic use, plain)
D55	Anaemia due to enzyme disorders		
D56	Thalassaemia		
D57	Sickle-cell disorders		
D58	Other hereditary haemolytic anaemias		
D59	Acquired haemolytic anaemia		
D60	Acquired pure red cell aplasia [erythroblastopenia]		
D61	Other aplastic anaemias		
D63	Anaemia in chronic diseases classified elsewhere		
D64	Other anaemias		

ASTHMA

ICD-10 codes and labels included in chronic disease category		*ATC-5 codes and labels for drugs associated to chronic disease category. Must include:*	
J45	Asthma	R03AC	Selective beta-2-adrenoreceptor agonists
		And at least one of the following:	

R03AK	Adrenergics in combination with corticosteroids or other drugs, excl. anticholinergics
R03BA	Glucocorticoids (inhalation)
R03BB	Anticholinergics

**ATRIAL FIBRILLATION**			
ICD-10 codes and labels included in chronic disease category		*ATC-5 codes and labels for drugs associated to chronic disease category. Includes none or any of the following:*	
I48	Atrial fibrillation and flutter	B01AA	Vitamin K antagonists
		C01AA	Digitalis glycosides
		C01BD	Antiarrhythmics, class III
		C07AB	Beta blocking agents, selective

**AUTOIMMUNE DISEASES**			
ICD-10 codes and labels included in chronic disease category		*ATC-5 codes and labels for drugs associated to chronic disease category. Must include at least one of the following:*	
I731	Thromboangiitis obliterans [Buerger]	D07AC	Corticosteroids, potent (group III) (topical use)
L10	Pemphigus	H02AB	Glucocorticoids (systemic use, plain)
L12	Pemphigoid	M01AX	Other antiinflammatory and antirheumatic agents, non-steroids
L40	Psoriasis	N02BE	Anilides
L41	Parapsoriasis		
L93	Lupus erythematosus		
L94	Other localized connective tissue disorders		
L95	Vasculitis limited to skin, not elsewhere classified		
M30	Polyarteritis nodosa and related conditions		
M31	Other necrotizing vasculopathies		
M32	Systemic lupus erythematosus		
M33	Dermatopolymyositis		
M34	Systemic sclerosis		
M35	Other systemic involvement of connective tissue		
M36	Systemic disorders of connective tissue in diseases classified elsewhere		

**BLINDNESS, VISUAL IMPAIRMENT**			
ICD-10 codes and labels included in chronic disease category		*ATC-5 codes and labels for drugs associated to chronic disease category. Includes none or any of the following:*	
H54	Visual impairment including blindness (binocular or monocular)	S01ED	Beta blocking agents
Z442	Fitting and adjustment of artificial eye		

Z970	Presence of artificial eye

**BLOOD AND BLOOD FORMING ORGAN DISEASES**			
ICD-10 codes and labels included in chronic disease category		*ATC-5 codes and labels for drugs associated to chronic disease category. Includes none or any of the following:*	
D66	Hereditary factor VIII deficiency	B01AA	Vitamin K antagonists
D67	Hereditary factor IX deficiency	B01AC	Platelet aggregation inhibitors excl. heparin
D68	Other coagulation defects	H02AB	Glucocorticoids (systemic use, plain)
D69	Purpura and other haemorrhagic conditions		
D71	Functional disorders of polymorphonuclear neutrophils		
D720	Genetic anomalies of leukocytes		
D730	Hyposplenism		
D731	Hypersplenism		
D732	Chronic congestive splenomegaly		
D74	Methaemoglobinaemia		
D750	Familial erythrocytosis		
D761	Haemophagocytic lymphohistiocytosis		
D763	Other histiocytosis syndromes		
D77	Other disorders of blood and blood-forming organs in diseases classified elsewhere		
D80	Immunodeficiency with predominantly antibody defects		
D81	Combined immunodeficiencies		
D82	Immunodeficiency associated with other major defects		
D83	Common variable immunodeficiency		
D84	Other immunodeficiencies		
D86	Sarcoidosis		
D89	Other disorders involving the immune mechanism, not elsewhere classified		

**BRADYCARDIAS AND CONDUCTION DISEASES**		
ICD-10 codes and labels included in chronic disease category		*Non-pharmacological treatment or treatment with drugs excluded from this study*
I441	Atrioventricular block, second degree	
I442	Atrioventricular block, complete	
I443	Other and unspecified atrioventricular block	
I453	Trifascicular block	
I455	Other specified heart block	

Z950	Presence of cardiac pacemaker

**CARDIAC VALVE DISEASES**			
ICD-10 codes and labels included in chronic disease category		*ATC-5 codes and labels for drugs associated to chronic disease category. Must include at least one of the following:*	
I05	Rheumatic mitral valve diseases	B01AA	Vitamin K antagonists
I06	Rheumatic aortic valve diseases		
I07	Rheumatic tricuspid valve diseases		
I08	Multiple valve diseases		
I091	Rheumatic diseases of endocardium, valve unspecified		
I098	Other specified rheumatic heart diseases		
I34	Nonrheumatic mitral valve disorders		
I35	Nonrheumatic aortic valve disorders		
I36	Nonrheumatic tricuspid valve disorders		
I37	Pulmonary valve disorders		
I38	Endocarditis, valve unspecified		
I390	Mitral valve disorders in diseases classified elsewhere		
I391	Aortic valve disorders in diseases classified elsewhere		
I392	Tricuspid valve disorders in diseases classified elsewhere		
I393	Pulmonary valve disorders in diseases classified elsewhere		
I394	Multiple valve disorders in diseases classified elsewhere		
Q22	Congenital malformations of pulmonary and tricuspid valves		
Q23	Congenital malformations of aortic and mitral valves		
Z952	Presence of prosthetic heart valve		
Z953	Presence of xenogenic heart valve		
Z954	Presence of other heart-valve replacement		

**CATARACT AND OTHER LENS DISEASES**		
ICD-10 codes and labels included in chronic disease category		*Non-pharmacological treatment or treatment with drugs excluded from this study*
H25	Senile cataract	
H26	Other cataract	
H27	Other disorders of lens	
H28	Cataract and other disorders of lens in diseases classified elsewhere	
Q12	Congenital lens malformations	
Z961	Presence of intraocular lens	

**CEREBROVASCULAR DISEASE**			
ICD-10 codes and labels included in chronic disease category		*ATC-5 codes and labels for drugs associated to chronic disease category. Includes none or any of the following:*	
G45	Transient cerebral ischaemic attacks and related syndromes	B01AC	Platelet aggregation inhibitors excl. heparin
G46	Vascular syndromes of brain in cerebrovascular diseases	C08CA	Dihydropyridine derivatives
I60	Subarachnoid haemorrhage		
I61	Intracerebral haemorrhage		
I62	Other nontraumatic intracranial haemorrhage		
I63	Cerebral infarction		
I64	Stroke, not specified as haemorrhage or infarction		
I67	Other cerebrovascular diseases		
I69	Sequelae of cerebrovascular disease		

**CHRONIC KIDNEY DISEASES**			
ICD-10 codes and labels included in chronic disease category		*ATC-5 codes and labels for drugs associated to chronic disease category. Must include at least one of the following:*	
I120	Hypertensive renal disease with renal failure	C03BA	Sulfonamides, plain
I130	Hypertensive heart and renal disease with (congestive) heart failure	C03CA	Sulfonamides, plain
I131	Hypertensive heart and renal disease with renal failure	C03EA	Low-ceiling diuretics and potassium-sparing agents
I132	Hypertensive heart and renal disease with both (congestive) heart failure and renal failure	C07AA	Beta blocking agents, non-selective
I139	Hypertensive heart and renal disease, unspecified	C07AB	Beta blocking agents, selective
N01	Rapidly progressive nephritic syndrome	C09AA	ACE inhibitors, plain
N03	Chronic nephritic syndrome	C09CA	Angiotensin II antagonists, plain
N04	Nephrotic syndrome	H02AB	Glucocorticoids (systemic use, plain)
N05	Unspecified nephritic syndrome	M01AX	Other antiinflammatory and antirheumatic agents, non-steroids
N07	Hereditary nephropathy, not elsewhere classified		
N08	Glomerular disorders in diseases classified elsewhere		
N11	Chronic tubulo-interstitial nephritis		
N183	Chronic kidney disease, stage 3		
N184	Chronic kidney disease, stage 4		
N185	Chronic kidney disease, stage 5		
N189	Chronic kidney disease, unspecified		
Q60	Renal agenesis and other reduction defects of kidney		
Q611	Polycystic kidney, autosomal recessive		

Q612	Polycystic kidney, autosomal dominant
Q613	Polycystic kidney, unspecified
Q614	Renal dysplasia
Q615	Medullary cystic kidney
Q618	Other cystic kidney diseases
Q619	Cystic kidney disease, unspecified
Z905	Acquired absence of kidney
Z940	Kidney transplant status

**CHRONIC LIVER DISEASES**			
ICD-10 codes and labels included in chronic disease category		*ATC-5 codes and labels for drugs associated to chronic disease category. Includes none or any of the following:*	
B18	Chronic viral hepatitis	C03AA	Thiazides, plain
K70	Alcoholic liver disease	C03BA	Sulfonamides, plain
K713	Toxic liver disease with chronic persistent hepatitis	C03CA	Sulfonamides, plain
K714	Toxic liver disease with chronic lobular hepatitis	C03DA	Aldosterone antagonists
K715	Toxic liver disease with chronic active hepatitis	C07AA	Beta blocking agents, non-selective
K717	Toxic liver disease with fibrosis and cirrhosis of liver	H02AB	Glucocorticoids (systemic use, plain)
K721	Chronic hepatic failure	M01AX	Other antiinflammatory and antirheumatic agents, non-steroids
K73	Chronic hepatitis, not elsewhere classified		
K74	Fibrosis and cirrhosis of liver		
K753	Granulomatous hepatitis, not elsewhere classified		
K754	Autoimmune hepatitis		
K758	Other specified inflammatory liver diseases		
K761	Chronic passive congestion of liver		
K766	Portal hypertension		
K767	Hepatorenal syndrome		
K778	Liver disorders in other diseases classified elsewhere		
Q446	Cystic disease of liver		
Z944	Liver transplant status		

**CHRONIC PANCREAS, BILIARY TRACT AND GALLBLADDER DISEASES**		
ICD-10 codes and labels included in chronic disease category		*Non-pharmacological treatment or treatment with drugs excluded from this study*
K800	Calculus of gallbladder with acute cholecystitis	
K801	Calculus of gallbladder with other cholecystitis	

K802	Calculus of gallbladder without cholecystitis
K808	Other cholelithiasis
K811	Chronic cholecystitis
K86	Other diseases of pancreas
Q440	Agenesis, aplasia and hypoplasia of gallbladder
Q441	Other congenital malformations of gallbladder
Q442	Atresia of bile ducts
Q443	Congenital stenosis and stricture of bile ducts
Q444	Choledochal cyst
Q445	Other congenital malformations of bile ducts
Q450	Agenesis, aplasia and hypoplasia of pancreas

**CHRONIC ULCER OF THE SKIN**			
ICD-10 codes and labels included in chronic disease category		*ATC-5 codes and labels for drugs associated to chronic disease category. Includes none or any of the following:*	
I830	Varicose veins of lower extremities with ulcer	C04AD	Purine derivatives
I832	Varicose veins of lower extremities with both ulcer and inflammation	M01AX	Other antiinflammatory and antirheumatic agents, non-steroids
L89	Decubitus ulcer and pressure area	N02AX	Other opioids
L97	Ulcer of lower limb, not elsewhere classified		
L984	Chronic ulcer of skin, not elsewhere classified		

**COLITIS AND RELATED DISEASES**			
ICD-10 codes and labels included in chronic disease category		*ATC-5 codes and labels for drugs associated to chronic disease category. Includes none or any of the following:*	
K520	Gastroenteritis and colitis due to radiation	B01AC	Platelet aggregation inhibitors excl. heparin
K528	Other specified noninfective gastroenteritis and colitis	B03AA	Iron bivalent, oral preparations
K551	Chronic vascular disorders of intestine	C01DA	Organic nitrates
K552	Angiodysplasia of colon	H02AB	Glucocorticoids (systemic use, plain)
K572	Diverticular disease of large intestine with perforation and abscess	M01AB	Acetic acid derivatives and related substances
K573	Diverticular disease of large intestine without perforation or abscess	M01AE	Propionic acid derivatives
K574	Diverticular disease of both small and large intestine with perforation and abscess	M01AH	Coxibs
K575	Diverticular disease of both small and large intestine without perforation or abscess	M01AX	Other antiinflammatory and antirheumatic agents, non-steroids
K578	Diverticular disease of intestine, part unspecified, with perforation and abscess	N02AB	Phenylpiperidine derivatives

K579	Diverticular disease of intestine, part unspecified, without perforation or abscess	N02AX	Other opioids
K58	Irritable bowel syndrome	N02BB	Pyrazolones
K590	Constipation	N02BE	Anilides
K592	Neurogenic bowel, not elsewhere classified	N06AA	Non-selective monoamine reuptake inhibitors
K62	Other diseases of anus and rectum		
K634	Enteroptosis		
K64	Haemorrhoids and perianal venous thrombosis		

**COPD, EMPHYSEMA, CHRONIC BRONCHITIS**			
ICD-10 codes and labels included in chronic disease category		*ATC-5 codes and labels for drugs associated to chronic disease category. Includes none or any of the following:*	
J41	Simple and mucopurulent chronic bronchitis	J01CR	Combinations of penicillins, incl. beta-lactamase inhibitors
J42	Unspecified chronic bronchitis	J01MA	Fluoroquinolones
J43	Emphysema	R03AC	Selective beta-2-adrenoreceptor agonists
J44	Other chronic obstructive pulmonary disease	R03AK	Adrenergics in combination with corticosteroids or other drugs, excl. anticholinergics
J47	Bronchiectasis	R03BA	Glucocorticoids (inhalation)
		R03BB	Anticholinergics

**DEAFNESS, HEARING IMPAIRMENT**			
ICD-10 codes and labels included in chronic disease category		*Non-pharmacological treatment or treatment with drugs excluded from this study*	
H80	Otosclerosis		
H90	Conductive and sensorineural hearing loss		
H911	Presbycusis		
H913	Deaf mutism, not elsewhere classified		
H919	Hearing loss, unspecified		
Q16	Congenital malformations of ear causing impairment of hearing		
Z453	Adjustment and management of implanted hearing device		
Z461	Fitting and adjustment of hearing aid		
Z962	Presence of otological and audiological implants		
Z974	Presence of external hearing-aid		

**DEMENTIA**			
ICD-10 codes and labels included in chronic disease category		*ATC-5 codes and labels for drugs associated to chronic disease category. Includes none or any of the following:*	
F00	Dementia in Alzheimer disease	N05AH	Diazepines, oxazepines, thiazepines and oxepines
F01	Vascular dementia	N05AX	Other antipsychotics

F02	Dementia in other diseases classified elsewhere	N06DA	Anticholinesterases
F03	Unspecified dementia	N06DX	Other anti-dementia drugs
F051	Delirium superimposed on dementia		
G30	Alzheimer disease		
G31	Other degenerative diseases of nervous system, not elsewhere classified		

**DEPRESSION AND MOOD DISEASES**			
ICD-10 codes and labels included in chronic disease category		*ATC-5 codes and labels for drugs associated to chronic disease category. Must include at least one of the following:*	
F30	Manic episode	N03AX	Other antiepileptics
F31	Bipolar affective disorder	N05AH	Diazepines, oxazepines, thiazepines and oxepines
F32	Depressive episode	N05AX	Other antipsychotics
F33	Recurrent depressive disorder	N05BA	Benzodiazepine derivatives
F34	Persistent mood [affective] disorders	N05CD	Benzodiazepine derivatives
F38	Other mood [affective] disorders	N05CF	Benzodiazepine related drugs
F39	Unspecified mood [affective] disorder	N06AA	Non-selective monoamine reuptake inhibitors
F412	Mixed anxiety and depressive disorder	N06AB	Selective serotonin reuptake inhibitors
		N06AX	Other antidepressants

**DIABETES**			
ICD-10 codes and labels included in chronic disease category		*ATC-5 codes and labels for drugs associated to chronic disease category. Includes none or any of the following:*	
E10	Insulin-dependent diabetes mellitus	A10AC	Insulins and analogues for injection, intermediate-acting
E11	Non-insulin-dependent diabetes mellitus	A10AD	Insulins and analogues for injection, intermediate- or long-acting combined with fast-acting
E13	Other specified diabetes mellitus	A10AE	Insulins and analogues for injection, long-acting
E14	Unspecified diabetes mellitus	A10BA	Biguanides
E891	Postprocedural hypoinsulinaemia	A10BB	Sulfonylureas
		A10BD	Combinations of oral blood glucose lowering drugs
		A10BX	Other blood glucose lowering drugs, excl. insulins

**DORSOPATHIES**			
ICD-10 codes and labels included in chronic disease category		*ATC-5 codes and labels for drugs associated to chronic disease category. Includes none or any of the following:*	
M40	Kyphosis and lordosis	H02AB	Glucocorticoids (systemic use, plain)
M41	Scoliosis	M01AB	Acetic acid derivatives and related substances
M42	Spinal osteochondrosis	M01AE	Propionic acid derivatives

M43	Other deforming dorsopathies	M01AH	Coxibs
M47	Spondylosis	M01AX	Other antiinflammatory and antirheumatic agents, non-steroids
M48	Other spondylopathies	M05BA	Bisphosphonates
M49	Spondylopathies in diseases classified elsewhere	N02AB	Phenylpiperidine derivatives
M50	Cervical disc disorders	N02AX	Other opioids
M51	Other intervertebral disc disorders	N02BB	Pyrazolones
M53	Other dorsopathies, not elsewhere classified	N02BE	Anilides
Q675	Congenital deformity of spine		
Q761	Klippel-Feil syndrome		
Q764	Other congenital malformations of spine, not associated with scoliosis		

**DYSLIPIDEMIA**			
ICD-10 codes and labels included in chronic disease category		*ATC-5 codes and labels for drugs associated to chronic disease category. Includes none or any of the following:*	
E78	Disorders of lipoprotein metabolism and other lipidaemias	C10AA	HMG CoA reductase inhibitors
		C10AB	Fibrates
		C10AX	Other lipid modifying agents

**EAR, NOSE, THROAT DISEASES**			
ICD-10 codes and labels included in chronic disease category		*ATC-5 codes and labels for drugs associated to chronic disease category. Includes none or any of the following:*	
H604	Cholesteatoma of external ear	H02AB	Glucocorticoids (systemic use, plain)
H661	Chronic tubotympanic suppurative otitis media	N07CA	Antivertigo preparations
H662	Chronic atticoantral suppurative otitis media	R01AD	Corticosteroids (nasal use)
H663	Other chronic suppurative otitis media	R06AX	Other antihistamines for systemic use
H701	Chronic mastoiditis		
H71	Cholesteatoma of middle ear		
H731	Chronic myringitis		
H741	Adhesive middle ear disease		
H810	MÚniÞre disease		
H831	Labyrinthine fistula		
H832	Labyrinthine dysfunction		
H95	Postprocedural disorders of ear and mastoid process, not elsewhere classified		
J300	Vasomotor rhinitis		
J31	Chronic rhinitis, nasopharyngitis and pharyngitis		

J32	Chronic sinusitis
J33	Nasal polyp
J341	Cyst and mucocele of nose and nasal sinus
J342	Deviated nasal septum
J343	Hypertrophy of nasal turbinates
J35	Chronic diseases of tonsils and adenoids
J37	Chronic laryngitis and laryngotracheitis
J380	Paralysis of vocal cords and larynx
J386	Stenosis of larynx
K051	Chronic gingivitis
K053	Chronic periodontitis
K07	Dentofacial anomalies [including malocclusion]
K110	Atrophy of salivary gland
K117	Disturbances of salivary secretion
Q30	Congenital malformations of nose
Q31	Congenital malformations of larynx
Q32	Congenital malformations of trachea and bronchus
Q35	Cleft palate
Q36	Cleft lip
Q37	Cleft palate with cleft lip
Q38	Other congenital malformations of tongue, mouth and pharynx

**EPILEPSY**			
ICD-10 codes and labels included in chronic disease category		*ATC-5 codes and labels for drugs associated to chronic disease category. Must include at least one of the following:*	
G40	Epilepsy	N03AE	Benzodiazepine derivatives
		N03AX	Other antiepileptics

**ESOPHAGUS, STOMACH AND DUODENUM DISEASES**			
ICD-10 codes and labels included in chronic disease category		*ATC-5 codes and labels for drugs associated to chronic disease category. Includes none or any of the following:*	
I85	Oesophageal varices	A02BA	H2-receptor antagonists
I864	Gastric varices	A02BC	Proton pump inhibitors
I982	Oesophageal varices without bleeding in diseases classified elsewhere	A03FA	Propulsives
I983	Oesophageal varices with bleeding in diseases classified elsewhere	A11CC	Vitamin D and analogues

K21	Gastro-oesophageal reflux disease	A12AA	Calcium
K220	Achalasia of cardia	A12AX	Calcium, combinations with vitamin D and/or other drugs
K222	Oesophageal obstruction	B03AA	Iron bivalent, oral preparations
K224	Dyskinesia of oesophagus	B03AB	Iron trivalent, oral preparations
K225	Diverticulum of oesophagus, acquired	B03BA	Vitamin B12 (cyanocobalamin and analogues)
K227	Barrett oesophagus	C01DA	Organic nitrates
K230	Tuberculous oesophagitis	C07AA	Beta blocking agents, non-selective
K231	Megaoesophagus in Chagas disease	C07AG	Alpha and beta blocking agents
K254	Gastric ulcer: Chronic or unspecified with haemorrhage	C08CA	Dihydropyridine derivatives
K255	Gastric ulcer: Chronic or unspecified with perforation	C08DB	Benzothiazepine derivatives
K256	Gastric ulcer: Chronic or unspecified with both haemorrhage and perforation		
K257	Gastric ulcer: Chronic without haemorrhage or perforation		
K264	Duodenal ulcer: Chronic or unspecified with haemorrhage		
K265	Duodenal ulcer: Chronic or unspecified with perforation		
K266	Duodenal ulcer: Chronic or unspecified with both haemorrhage and perforation		
K267	Duodenal ulcer: Chronic without haemorrhage or perforation		
K274	Peptic ulcer, site unspecified: Chronic or unspecified with haemorrhage		
K275	Peptic ulcer, site unspecified: Chronic or unspecified with perforation		
K276	Peptic ulcer, site unspecified: Chronic or unspecified with both haemorrhage and perforation		
K277	Peptic ulcer, site unspecified: Chronic without haemorrhage or perforation		
K284	Gastrojejunal ulcer: Chronic or unspecified with haemorrhage		
K285	Gastrojejunal ulcer: Chronic or unspecified with perforation		
K286	Gastrojejunal ulcer: Chronic or unspecified with both haemorrhage and perforation		
K287	Gastrojejunal ulcer: Chronic without haemorrhage or perforation		
K293	Chronic superficial gastritis		
K294	Chronic atrophic gastritis		
K295	Chronic gastritis, unspecified		
K296	Other gastritis		
K297	Gastritis, unspecified		
K298	Duodenitis		

K299	Gastroduodenitis, unspecified
K311	Adult hypertrophic pyloric stenosis
K312	Hourglass stricture and stenosis of stomach
K313	Pylorospasm, not elsewhere classified
K314	Gastric diverticulum
K315	Obstruction of duodenum
Q39	Congenital malformations of oesophagus
Q40	Other congenital malformations of upper alimentary tract
Z903	Acquired absence of part of stomach

**GLAUCOMA**			
ICD-10 codes and labels included in chronic disease category		*ATC-5 codes and labels for drugs associated to chronic disease category. Includes none or any of the following:*	
H401	Primary open-angle glaucoma	S01EC	Carbonic anhydrase inhibitors
H402	Primary angle-closure glaucoma	S01ED	Beta blocking agents
H403	Glaucoma secondary to eye trauma	S01EE	Prostaglandin analogues
H404	Glaucoma secondary to eye inflammation		
H405	Glaucoma secondary to other eye disorders		
H406	Glaucoma secondary to drugs		
H408	Other glaucoma		
H409	Glaucoma, unspecified		

**HEART FAILURE**			
ICD-10 codes and labels included in chronic disease category		*ATC-5 codes and labels for drugs associated to chronic disease category. Includes none or any of the following:*	
I110	Hypertensive heart disease with (congestive) heart failure	A11CC	Vitamin D and analogues
I130	Hypertensive heart and renal disease with (congestive) heart failure	A12AA	Calcium
I132	Hypertensive heart and renal disease with both (congestive) heart failure and renal failure	B01AA	Vitamin K antagonists
I27	Other pulmonary heart diseases	B01AC	Platelet aggregation inhibitors excl. heparin
I280	Arteriovenous fistula of pulmonary vessels	B03AA	Iron bivalent, oral preparations
I42	Cardiomyopathy	B03AB	Iron trivalent, oral preparations
I43	Cardiomyopathy in diseases classified elsewhere	C01AA	Digitalis glycosides
I50	Heart failure	C01BD	Antiarrhythmics, class III
I515	Myocardial degeneration	C01DA	Organic nitrates
I517	Cardiomegaly	C01EB	Other cardiac preparations

I528	Other heart disorders in other diseases classified elsewhere	C03AA	Thiazides, plain
Z941	Heart transplant status	C03BA	Sulfonamides, plain
Z943	Heart and lungs transplant status	C03CA	Sulfonamides, plain
		C03DA	Aldosterone antagonists
		C03EA	Low-ceiling diuretics and potassium-sparing agents
		C07AA	Beta blocking agents, non-selective
		C07AB	Beta blocking agents, selective
		C07AG	Alpha and beta blocking agents
		C08CA	Dihydropyridine derivatives
		C08DB	Benzothiazepine derivatives
		C09AA	ACE inhibitors, plain
		C09BA	ACE inhibitors and diuretics
		C09CA	Angiotensin II antagonists, plain
		C09DA	Angiotensin II antagonists and diuretics
		C09DB	Angiotensin II antagonists and calcium channel blockers

**HYPERTENSION**			
ICD-10 codes and labels included in chronic disease category		*ATC-5 codes and labels for drugs associated to chronic disease category. Includes none or any of the following:*	
I10	Essential (primary) hypertension	C02CA	Alpha-adrenoreceptor antagonists
I11	Hypertensive heart disease	C03AA	Thiazides, plain
I12	Hypertensive renal disease	C03BA	Sulfonamides, plain
I13	Hypertensive heart and renal disease	C03CA	Sulfonamides, plain
I15	Secondary hypertension	C03DA	Aldosterone antagonists
		C03EA	Low-ceiling diuretics and potassium-sparing agents
		C07AA	Beta blocking agents, non-selective
		C07AB	Beta blocking agents, selective
		C07AG	Alpha and beta blocking agents
		C08CA	Dihydropyridine derivatives
		C08DB	Benzothiazepine derivatives
		C09AA	ACE inhibitors, plain
		C09BA	ACE inhibitors and diuretics
		C09CA	Angiotensin II antagonists, plain
		C09DA	Angiotensin II antagonists and diuretics
		C09DB	Angiotensin II antagonists and calcium channel blockers

INFLAMMATORY ARTHROPATHIES

ICD-10 codes and labels included in chronic disease category		*ATC-5 codes and labels for drugs associated to chronic disease category. Includes none or any of the following:*	
M023	Reiter disease	H02AB	Glucocorticoids (systemic use, plain)
M05	Seropositive rheumatoid arthritis	M01AB	Acetic acid derivatives and related substances
M06	Other rheumatoid arthritis	M01AE	Propionic acid derivatives
M07	Psoriatic and enteropathic arthropathies	M01AH	Coxibs
M08	Juvenile arthritis	M01AX	Other antiinflammatory and antirheumatic agents, non-steroids
M09	Juvenile arthritis in diseases classified elsewhere	M04AA	Preparations inhibiting uric acid production
M10	Gout		
M11	Other crystal arthropathies		
M12	Other specific arthropathies		
M13	Other arthritis		
M14	Arthropathies in other diseases classified elsewhere		
M45	Ankylosing spondylitis		
M460	Spinal enthesopathy		
M461	Sacroiliitis, not elsewhere classified		
M468	Other specified inflammatory spondylopathies		
M469	Inflammatory spondylopathy, unspecified		

ISCHEMIC HEART DISEASE

ICD-10 codes and labels included in chronic disease category		*ATC-5 codes and labels for drugs associated to chronic disease category. Must include:*	
I20	Angina pectoris	B01AC	Platelet aggregation inhibitors excl. heparin
I21	Acute myocardial infarction	*And at least one of the following:*	
I22	Subsequent myocardial infarction	C01DA	Organic nitrates
I24	Other acute ischaemic heart diseases	C01EB	Other cardiac preparations
I25	Chronic ischaemic heart disease	C07AA	Beta blocking agents, non-selective
Z951	Presence of aortocoronary bypass graft	C07AB	Beta blocking agents, selective
Z955	Presence of coronary angioplasty implant and graft	C07AG	Alpha and beta blocking agents
		C08CA	Dihydropyridine derivatives
		C08DB	Benzothiazepine derivatives
		C09AA	ACE inhibitors, plain
		C09CA	Angiotensin II antagonists, plain
		C10AA	HMG CoA reductase inhibitors

		C10AX	Other lipid modifying agents

MIGRAINE AND FACIAL PAIN SYNDROMES			
ICD-10 codes and labels included in chronic disease category		*ATC-5 codes and labels for drugs associated to chronic disease category. Includes none or any of the following:*	
G43	Migraine	B03BA	Vitamin B12 (cyanocobalamin and analogues)
G440	Cluster headache syndrome	C07AA	Beta blocking agents, non-selective
G441	Vascular headache, not elsewhere classified	C07AB	Beta blocking agents, selective
G442	Tension-type headache	M01AB	Acetic acid derivatives and related substances
G443	Chronic post-traumatic headache	M01AE	Propionic acid derivatives
G448	Other specified headache syndromes	M01AH	Coxibs
G50	Disorders of trigeminal nerve	N03AX	Other antiepileptics
		N06AA	Non-selective monoamine reuptake inhibitors
		N07CA	Antivertigo preparations

NEUROTIC, STRESS-RELATED AND SOMATOFORM DISEASES			
ICD-10 codes and labels included in chronic disease category		*ATC-5 codes and labels for drugs associated to chronic disease category. Includes none or any of the following:*	
F40	Phobic anxiety disorders	C07AA	Beta blocking agents, non-selective
F41	Other anxiety disorders	N02AB	Phenylpiperidine derivatives
F42	Obsessive-compulsive disorder	N02AX	Other opioids
F43	Reaction to severe stress, and adjustment disorders	N02BB	Pyrazolones
F44	Dissociative [conversion] disorders	N02BE	Anilides
F45	Somatoform disorders	N03AX	Other antiepileptics
F48	Other neurotic disorders	N05AH	Diazepines, oxazepines, thiazepines and oxepines
		N05BA	Benzodiazepine derivatives
		N06AA	Non-selective monoamine reuptake inhibitors
		N06AB	Selective serotonin reuptake inhibitors

OBESITY			
ICD-10 codes and labels included in chronic disease category		*Non-pharmacological treatment or treatment with drugs excluded from this study*	
E66	Obesity		

OSTEOARTHRITIS AND OTHER DEGENERATIVE JOINT DISEASES

ICD-10 codes and labels included in chronic disease category		ATC-5 codes and labels for drugs associated to chronic disease category. Includes none or any of the following:	
M15	Polyarthrosis	H02AB	Glucocorticoids (systemic use, plain)
M16	Coxarthrosis [arthrosis of hip]	M01AB	Acetic acid derivatives and related substances
M17	Gonarthrosis [arthrosis of knee]	M01AE	Propionic acid derivatives
M18	Arthrosis of first carpometacarpal joint	M01AH	Coxibs
M19	Other arthrosis	M01AX	Other antiinflammatory and antirheumatic agents, non-steroids
M362	Haemophilic arthropathy	N02AB	Phenylpiperidine derivatives
M363	Arthropathy in other blood disorders	N02AX	Other opioids
		N02BB	Pyrazolones
		N02BE	Anilides

OSTEOPOROSIS

ICD-10 codes and labels included in chronic disease category		ATC-5 codes and labels for drugs associated to chronic disease category. Includes none or any of the following:	
M80	Osteoporosis with pathological fracture	A11CC	Vitamin D and analogues
M81	Osteoporosis without pathological fracture	A12AA	Calcium
M82	Osteoporosis in diseases classified elsewhere	A12AX	Calcium, combinations with vitamin D and/or other drugs
		M05BA	Bisphosphonates
		M05BX	Other drugs affecting bone structure and mineralization

OTHER DIGESTIVE DISEASES

ICD-10 codes and labels included in chronic disease category		Non-pharmacological treatment or treatment with drugs excluded from this study
K660	Peritoneal adhesions	
K900	Celiac disease	
K901	Tropical sprue	
K902	Blind loop syndrome, not elsewhere classified	
K911	Postgastric surgery syndromes	
K93	Disorders of other digestive organs in diseases classified elsewhere	
Q41	Congenital absence, atresia and stenosis of small intestine	
Q42	Congenital absence, atresia and stenosis of large intestine	
Q43	Other congenital malformations of intestine	
R15	Faecal incontinence	
Z904	Acquired absence of other parts of digestive tract	
Z980	Intestinal bypass and anastomosis status	

OTHER EYE DISEASES			
ICD-10 codes and labels included in chronic disease category		*ATC-5 codes and labels for drugs associated to chronic disease category. Includes none or any of the following:*	
H022	Lagophthalmos	H02AB	Glucocorticoids (systemic use, plain)
H023	Blepharochalasis		
H024	Ptosis of eyelid		
H025	Other disorders affecting eyelid function		
H04	Disorders of lacrimal system		
H05	Disorders of orbit		
H104	Chronic conjunctivitis		
H17	Corneal scars and opacities		
H184	Corneal degeneration		
H185	Hereditary corneal dystrophies		
H186	Keratoconus		
H187	Other corneal deformities		
H188	Other specified disorders of cornea		
H189	Disorder of cornea, unspecified		
H193	Keratitis and keratoconjunctivitis in other diseases classified elsewhere		
H198	Other disorders of sclera and cornea in diseases classified elsewhere		
H201	Chronic iridocyclitis		
H21	Other disorders of iris and ciliary body		
H310	Chorioretinal scars		
H311	Choroidal degeneration		
H312	Hereditary choroidal dystrophy		
H318	Other specified disorders of choroid		
H319	Disorder of choroid, unspecified		
H33	Retinal detachments and breaks		
H352	Other proliferative retinopathy		
H353	Degeneration of macula and posterior pole		
H354	Peripheral retinal degeneration		
H355	Hereditary retinal dystrophy		
H357	Separation of retinal layers		
H358	Other specified retinal disorders		

H359	Retinal disorder, unspecified
H36	Retinal disorders in diseases classified elsewhere
H47	Other disorders of optic [2nd] nerve and visual pathways
H48	Disorders of optic [2nd] nerve and visual pathways in diseases classified elsewhere
H49	Paralytic strabismus
H51	Other disorders of binocular movement
Q10	Congenital malformations of eyelid, lacrimal apparatus and orbit
Q11	Anophthalmos, microphthalmos and macrophthalmos
Q13	Congenital malformations of anterior segment of eye
Q14	Congenital malformations of posterior segment of eye
Q15	Other congenital malformations of eye
Z947	Corneal transplant status

**OTHER GENITOURINARY DISEASES**			
ICD-10 codes and labels included in chronic disease category		*ATC-5 codes and labels for drugs associated to chronic disease category. Includes none or any of the following:*	
B901	Sequelae of genitourinary tuberculosis	C02CA	Alpha-adrenoreceptor antagonists
N200	Calculus of kidney	G03CA	Natural and semisynthetic estrogens, plain
N202	Calculus of kidney with calculus of ureter	G04BD	Drugs for urinary frequency and incontinence
N209	Urinary calculus, unspecified	G04CA	Alpha-adrenoreceptor antagonists
N210	Calculus in bladder	J01CR	Combinations of penicillins, incl. beta-lactamase inhibitors
N218	Other lower urinary tract calculus	J01MA	Fluoroquinolones
N219	Calculus of lower urinary tract, unspecified	M01AB	Acetic acid derivatives and related substances
N22	Calculus of urinary tract in diseases classified elsewhere	M01AE	Propionic acid derivatives
N301	Interstitial cystitis (chronic)	M01AH	Coxibs
N302	Other chronic cystitis	M01AX	Other antiinflammatory and antirheumatic agents, non-steroids
N303	Trigonitis	N06AA	Non-selective monoamine reuptake inhibitors
N304	Irradiation cystitis		
N31	Neuromuscular dysfunction of bladder, not elsewhere classified		
N320	Bladder-neck obstruction		
N323	Diverticulum of bladder		
N328	Other specified disorders of bladder		
N329	Bladder disorder, unspecified		
N33	Bladder disorders in diseases classified elsewhere		

N35	Urethral stricture
N393	Stress incontinence
N394	Other specified urinary incontinence
N480	Leukoplakia of penis
N484	Impotence of organic origin
N489	Disorder of penis, unspecified
N701	Chronic salpingitis and oophoritis
N711	Chronic inflammatory disease of uterus
N731	Chronic parametritis and pelvic cellulitis
N734	Female chronic pelvic peritonitis
N736	Female pelvic peritoneal adhesions
N761	Subacute and chronic vaginitis
N763	Subacute and chronic vulvitis
N81	Female genital prolapse
N88	Other noninflammatory disorders of cervix uteri
N895	Stricture and atresia of vagina
N905	Atrophy of vulva
N952	Postmenopausal atrophic vaginitis
Q54	Hypospadias
Q620	Congenital hydronephrosis
Q621	Atresia and stenosis of ureter
Q622	Congenital megaloureter
Q623	Other obstructive defects of renal pelvis and ureter
Q624	Agenesis of ureter
Q627	Congenital vesico-uretero-renal reflux
Q628	Other congenital malformations of ureter
Q638	Other specified congenital malformations of kidney
Q639	Congenital malformation of kidney, unspecified
Q640	Epispadias
Q641	Exstrophy of urinary bladder
Q643	Other atresia and stenosis of urethra and bladder neck
Q644	Malformation of urachus
Q645	Congenital absence of bladder and urethra
Q646	Congenital diverticulum of bladder

Q647	Other congenital malformations of bladder and urethra
Q648	Other specified congenital malformations of urinary system
Q649	Congenital malformation of urinary system, unspecified
Z906	Acquired absence of other organs of urinary tract
Z907	Acquired absence of genital organ(s)
Z960	Presence of urogenital implants

**OTHER METABOLIC DISEASES**			
ICD-10 codes and labels included in chronic disease category		*ATC-5 codes and labels for drugs associated to chronic disease category. Includes none or any of the following:*	
E20	Hypoparathyroidism	A11CC	Vitamin D and analogues
E21	Hyperparathyroidism and other disorders of parathyroid gland	A12AA	Calcium
E22	Hyperfunction of pituitary gland	A12AX	Calcium, combinations with vitamin D and/or other drugs
E23	Hypofunction and other disorders of pituitary gland	B03AA	Iron bivalent, oral preparations
E24	Cushing syndrome	B03AB	Iron trivalent, oral preparations
E25	Adrenogenital disorders	B03BA	Vitamin B12 (cyanocobalamin and analogues)
E26	Hyperaldosteronism	C03AA	Thiazides, plain
E27	Other disorders of adrenal gland	C03BA	Sulfonamides, plain
E28	Ovarian dysfunction	C03CA	Sulfonamides, plain
E29	Testicular dysfunction	C03DA	Aldosterone antagonists
E31	Polyglandular dysfunction	G03CA	Natural and semisynthetic estrogens, plain
E34	Other endocrine disorders	H02AB	Glucocorticoids (systemic use, plain)
E35	Disorders of endocrine glands in diseases classified elsewhere	H03AA	Thyroid hormones
E40	Kwashiorkor	M04AA	Preparations inhibiting uric acid production
E41	Nutritional marasmus	M05BA	Bisphosphonates
E42	Marasmic kwashiorkor		
E43	Unspecified severe protein-energy malnutrition		
E44	Protein-energy malnutrition of moderate and mild degree		
E45	Retarded development following protein-energy malnutrition		
E46	Unspecified protein-energy malnutrition		
E64	Sequelae of malnutrition and other nutritional deficiencies		
E70	Disorders of aromatic amino-acid metabolism		
E71	Disorders of branched-chain amino-acid metabolism and fatty-acid metabolism		
E72	Other disorders of amino-acid metabolism		

E74	Other disorders of carbohydrate metabolism
E75	Disorders of sphingolipid metabolism and other lipid storage disorders
E76	Disorders of glycosaminoglycan metabolism
E77	Disorders of glycoprotein metabolism
E79	Disorders of purine and pyrimidine metabolism
E80	Disorders of porphyrin and bilirubin metabolism
E83	Disorders of mineral metabolism
E84	Cystic fibrosis
E85	Amyloidosis
E88	Other metabolic disorders
E89	Postprocedural endocrine and metabolic disorders, not elsewhere classified
K903	Pancreatic steatorrhoea
K904	Malabsorption due to intolerance, not elsewhere classified
K908	Other intestinal malabsorption
K909	Intestinal malabsorption, unspecified
K912	Postsurgical malabsorption, not elsewhere classified
M83	Adult osteomalacia
M88	Paget disease of bone [osteitis deformans]
N25	Disorders resulting from impaired renal tubular function

**OTHER MUSCULOSKELETAL AND JOINT DISEASES**			
ICD-10 codes and labels included in chronic disease category		*ATC-5 codes and labels for drugs associated to chronic disease category. Includes none or any of the following:*	
B902	Sequelae of tuberculosis of bones and joints	M01AB	Acetic acid derivatives and related substances
M212	Flexion deformity	M01AE	Propionic acid derivatives
M213	Wrist or foot drop (acquired)	M01AH	Coxibs
M214	Flat foot [pes planus] (acquired)	N02AB	Phenylpiperidine derivatives
M215	Acquired clawhand, clubhand, clawfoot and clubfoot	N02AX	Other opioids
M216	Other acquired deformities of ankle and foot	N02BB	Pyrazolones
M217	Unequal limb length (acquired)	N02BE	Anilides
M218	Other specified acquired deformities of limbs	N06AA	Non-selective monoamine reuptake inhibitors
M219	Acquired deformity of limb, unspecified	N06AB	Selective serotonin reuptake inhibitors
M22	Disorders of patella	N06AX	Other antidepressants
M23	Internal derangement of knee		

M24	Other specific joint derangements
M252	Flail joint
M253	Other instability of joint
M357	Hypermobility syndrome
M61	Calcification and ossification of muscle
M652	Calcific tendinitis
M653	Trigger finger
M654	Radial styloid tenosynovitis [de Quervain]
M700	Chronic crepitant synovitis of hand and wrist
M720	Palmar fascial fibromatosis [Dupuytren]
M722	Plantar fascial fibromatosis
M724	Pseudosarcomatous fibromatosis
M750	Adhesive capsulitis of shoulder
M751	Rotator cuff syndrome
M753	Calcific tendinitis of shoulder
M754	Impingement syndrome of shoulder
M797	Fibromyalgia
M841	Nonunion of fracture [pseudarthrosis]
M89	Other disorders of bone
M91	Juvenile osteochondrosis of hip and pelvis
M93	Other osteochondropathies
M94	Other disorders of cartilage
M96	Postprocedural musculoskeletal disorders, not elsewhere classified
M99	Biomechanical lesions, not elsewhere classified
Q65	Congenital deformities of hip
Q66	Congenital deformities of feet
Q68	Other congenital musculoskeletal deformities
Q71	Reduction defects of upper limb
Q72	Reduction defects of lower limb
Q73	Reduction defects of unspecified limb
Q74	Other congenital malformations of limb(s)
Q77	Osteochondrodysplasia with defects of growth of tubular bones and spine
Q78	Other osteochondrodysplasias
Q796	Ehlers-Danlos syndrome

Q798	Other congenital malformations of musculoskeletal system
Q87	Other specified congenital malformation syndromes affecting multiple systems
S382	Traumatic amputation of external genital organs
S48	Traumatic amputation of shoulder and upper arm
S58	Traumatic amputation of forearm
S68	Traumatic amputation of wrist and hand
S78	Traumatic amputation of hip and thigh
S88	Traumatic amputation of lower leg
S98	Traumatic amputation of ankle and foot
T05	Traumatic amputations involving multiple body regions
T096	Traumatic amputation of trunk, level unspecified
T116	Traumatic amputation of upper limb, level unspecified
T136	Traumatic amputation of lower limb, level unspecified
T147	Crushing injury and traumatic amputation of unspecified body region
T90	Sequelae of injuries of head
T91	Sequelae of injuries of neck and trunk
T92	Sequelae of injuries of upper limb
T93	Sequelae of injuries of lower limb
T94	Sequelae of injuries involving multiple and unspecified body regions
T95	Sequelae of burns, corrosions and frostbite
T96	Sequelae of poisoning by drugs, medicaments and biological substances
T97	Sequelae of toxic effects of substances chiefly nonmedicinal as to source
T98	Sequelae of other and unspecified effects of external causes
Z440	Fitting and adjustment of artificial arm (complete)(partial)
Z441	Fitting and adjustment of artificial leg (complete)(partial)
Z891	Acquired absence of hand and wrist
Z892	Acquired absence of upper limb above wrist
Z893	Acquired absence of both upper limbs [any level]
Z894	Acquired absence of foot and ankle
Z895	Acquired absence of leg at or below knee
Z896	Acquired absence of leg above knee

Z897	Acquired absence of both lower limbs [any level, except toes alone]
Z898	Acquired absence of upper and lower limbs [any level]
Z899	Acquired absence of limb, unspecified
Z946	Bone transplant status
Z966	Presence of orthopaedic joint implants
Z971	Presence of artificial limb (complete)(partial)

**OTHER NEUROLOGICAL DISEASES**			
ICD-10 codes and labels included in chronic disease category		*ATC-5 codes and labels for drugs associated to chronic disease category. Includes none or any of the following:*	
B900	Sequelae of central nervous system tuberculosis	C03DA	Aldosterone antagonists
D482	Neoplasm of uncertain or unknown behaviour: Peripheral nerves and autonomic nervous system	C07AA	Beta blocking agents, non-selective
G041	Tropical spastic paraplegia	H02AB	Glucocorticoids (systemic use, plain)
G09	Sequelae of inflammatory diseases of central nervous system	N02BE	Anilides
G10	Huntington disease	N03AE	Benzodiazepine derivatives
G11	Hereditary ataxia	N03AX	Other antiepileptics
G12	Spinal muscular atrophy and related syndromes	N05AH	Diazepines, oxazepines, thiazepines and oxepines
G13	Systemic atrophies primarily affecting central nervous system in diseases classified elsewhere	N05AX	Other antipsychotics
G24	Dystonia	N06AA	Non-selective monoamine reuptake inhibitors
G25	Other extrapyramidal and movement disorders	S01EC	Carbonic anhydrase inhibitors
G26	Extrapyramidal and movement disorders in diseases classified elsewhere		
G32	Other degenerative disorders of nervous system in diseases classified elsewhere		
G37	Other demyelinating diseases of central nervous system		
G51	Facial nerve disorders		
G52	Disorders of other cranial nerves		
G53	Cranial nerve disorders in diseases classified elsewhere		
G70	Myasthenia gravis and other myoneural disorders		
G71	Primary disorders of muscles		
G723	Periodic paralysis		
G724	Inflammatory myopathy, not elsewhere classified		
G728	Other specified myopathies		
G729	Myopathy, unspecified		

G73	Disorders of myoneural junction and muscle in diseases classified elsewhere
G80	Cerebral palsy
G81	Hemiplegia
G82	Paraplegia and tetraplegia
G83	Other paralytic syndromes
G90	Disorders of autonomic nervous system
G91	Hydrocephalus
G938	Other specified disorders of brain
G939	Disorder of brain, unspecified
G95	Other diseases of spinal cord
G99	Other disorders of nervous system in diseases classified elsewhere
M471	Other spondylosis with myelopathy
Q00	Anencephaly and similar malformations
Q01	Encephalocele
Q02	Microcephaly
Q03	Congenital hydrocephalus
Q04	Other congenital malformations of brain
Q05	Spina bifida
Q06	Other congenital malformations of spinal cord
Q07	Other congenital malformations of nervous system
Q760	Spina bifida occulta

**OTHER PSYCHIATRIC AND BEHAVIORAL DISEASES**			
ICD-10 codes and labels included in chronic disease category		*ATC-5 codes and labels for drugs associated to chronic disease category. Includes none or any of the following:*	
F04	Organic amnesic syndrome, not induced by alcohol and other psychoactive substances	N03AX	Other antiepileptics
F06	Other mental disorders due to brain damage and dysfunction and to physical disease	N05AH	Diazepines, oxazepines, thiazepines and oxepines
F07	Personality and behavioural disorders due to brain disease, damage and dysfunction	N05AX	Other antipsychotics
F09	Unspecified organic or symptomatic mental disorder	N05BA	Benzodiazepine derivatives
F102	Mental and behavioural disorders due to use of alcohol: Dependence syndrome	N05CD	Benzodiazepine derivatives
F106	Mental and behavioural disorders due to use of alcohol: Amnesic syndrome	N05CF	Benzodiazepine related drugs

F107	Mental and behavioural disorders due to use of alcohol: Residual and late-onset psychotic disorder	N06AA	Non-selective monoamine reuptake inhibitors
F112	Mental and behavioural disorders due to use of opioids: Dependence syndrome	N06AB	Selective serotonin reuptake inhibitors
F116	Mental and behavioural disorders due to use of opioids: Amnesic syndrome	N06AX	Other antidepressants
F117	Mental and behavioural disorders due to use of opioids: Residual and late-onset psychotic disorder		
F122	Mental and behavioural disorders due to use of cannabinoids: Dependence syndrome		
F126	Mental and behavioural disorders due to use of cannabinoids: Amnesic syndrome		
F127	Mental and behavioural disorders due to use of cannabinoids: Residual and late-onset psychotic disorder		
F132	Mental and behavioural disorders due to use of sedatives or hypnotics: Dependence syndrome		
F136	Mental and behavioural disorders due to use of sedatives or hypnotics: Amnesic syndrome		
F137	Mental and behavioural disorders due to use of sedatives or hypnotics: Residual and late-onset psychotic disorder		
F142	Mental and behavioural disorders due to use of cocaine: Dependence syndrome		
F146	Mental and behavioural disorders due to use of cocaine: Amnesic syndrome		
F147	Mental and behavioural disorders due to use of cocaine: Residual and late-onset psychotic disorder		
F152	Mental and behavioural disorders due to use of other stimulants, including caffeine: Dependence syndrome		
F156	Mental and behavioural disorders due to use of other stimulants, including caffeine: Amnesic syndrome		
F157	Mental and behavioural disorders due to use of other stimulants, including caffeine: Residual and late-onset psychotic disorder		
F162	Mental and behavioural disorders due to use of hallucinogens: Dependence syndrome		
F166	Mental and behavioural disorders due to use of hallucinogens: Amnesic syndrome		
F167	Mental and behavioural disorders due to use of hallucinogens: Residual and late-onset psychotic disorder		
F172	Mental and behavioural disorders due to use of tobacco: Dependence syndrome		
F176	Mental and behavioural disorders due to use of tobacco: Amnesic syndrome		

F177	Mental and behavioural disorders due to use of tobacco: Residual and late-onset psychotic disorder
F182	Mental and behavioural disorders due to use of volatile solvents: Dependence syndrome
F186	Mental and behavioural disorders due to use of volatile solvents: Amnesic syndrome
F187	Mental and behavioural disorders due to use of volatile solvents: Residual and late-onset psychotic disorder
F192	Mental and behavioural disorders due to multiple drug use and use of other psychoactive substances: Dependence syndrome
F196	Mental and behavioural disorders due to multiple drug use and use of other psychoactive substances: Amnesic syndrome
F197	Mental and behavioural disorders due to multiple drug use and use of other psychoactive substances: Residual and late-onset psychotic disorder
F50	Eating disorders
F52	Sexual dysfunction, not caused by organic disorder or disease
F60	Specific personality disorders
F61	Mixed and other personality disorders
F62	Enduring personality changes, not attributable to brain damage and disease
F63	Habit and impulse disorders
F68	Other disorders of adult personality and behaviour
F70	Mild mental retardation
F71	Moderate mental retardation
F72	Severe mental retardation
F73	Profound mental retardation
F78	Other mental retardation
F79	Unspecified mental retardation
F80	Specific developmental disorders of speech and language
F81	Specific developmental disorders of scholastic skills
F82	Specific developmental disorder of motor function
F83	Mixed specific developmental disorders
F84	Pervasive developmental disorders
F88	Other disorders of psychological development
F89	Unspecified disorder of psychological development
F95	Tic disorders
F99	Mental disorder, not otherwise specified

PARKINSON AND PARKINSONISM

ICD-10 codes and labels included in chronic disease category		*ATC-5 codes and labels for drugs associated to chronic disease category. Includes none or any of the following:*	
G20	Parkinson disease	N04BA	Dopa and dopa derivatives
G21	Secondary parkinsonism		
G22	Parkinsonism in diseases classified elsewhere		
G23	Other degenerative diseases of basal ganglia		

PERIPHERAL NEUROPATHY

ICD-10 codes and labels included in chronic disease category		*ATC-5 codes and labels for drugs associated to chronic disease category. Includes none or any of the following:*	
B91	Sequelae of poliomyelitis	H02AB	Glucocorticoids (systemic use, plain)
G14	Postpolio syndrome	M01AB	Acetic acid derivatives and related substances
G54	Nerve root and plexus disorders	M01AE	Propionic acid derivatives
G55	Nerve root and plexus compressions in diseases classified elsewhere	M01AH	Coxibs
G56	Mononeuropathies of upper limb	N02AB	Phenylpiperidine derivatives
G57	Mononeuropathies of lower limb	N02AX	Other opioids
G58	Other mononeuropathies	N02BB	Pyrazolones
G59	Mononeuropathy in diseases classified elsewhere	N02BE	Anilides
G60	Hereditary and idiopathic neuropathy	N03AX	Other antiepileptics
G628	Other specified polyneuropathies	N06AA	Non-selective monoamine reuptake inhibitors
G629	Polyneuropathy, unspecified		
G63	Polyneuropathy in diseases classified elsewhere		
M472	Other spondylosis with radiculopathy		
M531	Cervicobrachial syndrome		
M541	Radiculopathy		

PERIPHERAL VASCULAR DISEASE

ICD-10 codes and labels included in chronic disease category		*ATC-5 codes and labels for drugs associated to chronic disease category. Includes none or any of the following:*	
I702	Atherosclerosis of arteries of extremities	B01AC	Platelet aggregation inhibitors excl. heparin
I73	Other peripheral vascular diseases	C02CA	Alpha-adrenoreceptor antagonists
I792	Peripheral angiopathy in diseases classified elsewhere	C04AD	Purine derivatives
I798	Other disorders of arteries, arterioles and capillaries in diseases classified elsewhere	C08CA	Dihydropyridine derivatives

PROSTATE DISEASES			
ICD-10 codes and labels included in chronic disease category		*ATC-5 codes and labels for drugs associated to chronic disease category. Includes none or any of the following:*	
N40	Hyperplasia of prostate	G04CA	Alpha-adrenoreceptor antagonists
N411	Chronic prostatitis	G04CB	Testosterone-5-alpha reductase inhibitors
N418	Other inflammatory diseases of prostate	G04CX	Other drugs used in benign prostatic hypertrophy
		M01AE	Propionic acid derivatives

SCHIZOPHRENIA AND DELUSIONAL DISEASES			
ICD-10 codes and labels included in chronic disease category		*ATC-5 codes and labels for drugs associated to chronic disease category. Includes none or any of the following:*	
F20	Schizophrenia	N05AH	Diazepines, oxazepines, thiazepines and oxepines
F22	Persistent delusional disorders	N05AX	Other antipsychotics
F24	Induced delusional disorder		
F25	Schizoaffective disorders		
F28	Other nonorganic psychotic disorders		

SLEEP DISORDERS			
ICD-10 codes and labels included in chronic disease category		*ATC-5 codes and labels for drugs associated to chronic disease category. Includes none or any of the following:*	
F510	Nonorganic insomnia	N05BA	Benzodiazepine derivatives
F511	Nonorganic hypersomnia	N05CD	Benzodiazepine derivatives
F512	Nonorganic disorder of the sleep-wake schedule	N05CF	Benzodiazepine related drugs
F513	Sleepwalking [somnambulism]		
G47	Sleep disorders		

SOLID NEOPLASMS		
ICD-10 codes and labels included in chronic disease category		*Non-pharmacological treatment or treatment with drugs excluded from this study*
C	Malignant neoplasms	
D00	Carcinoma in situ of oral cavity, oesophagus and stomach	
D01	Carcinoma in situ of other and unspecified digestive organs	
D02	Carcinoma in situ of middle ear and respiratory system	
D03	Melanoma in situ	
D04	Carcinoma in situ of skin	
D05	Carcinoma in situ of breast	

D06	Carcinoma in situ of cervix uteri
D07	Carcinoma in situ of other and unspecified genital organs
D09	Carcinoma in situ of other and unspecified sites
D320	Benign neoplasm: Cerebral meninges
D321	Benign neoplasm: Spinal meninges
D329	Benign neoplasm: Meninges, unspecified
D330	Benign neoplasm: Brain, supratentorial
D331	Benign neoplasm: Brain, infratentorial
D332	Benign neoplasm: Brain, unspecified
D333	Benign neoplasm: Cranial nerves
D334	Benign neoplasm: Spinal cord
Q85	Phakomatoses, not elsewhere classified

**THYROID DISEASES**			
ICD-10 codes and labels included in chronic disease category		*ATC-5 codes and labels for drugs associated to chronic disease category. Includes none or any of the following:*	
E00	Congenital iodine-deficiency syndrome	H03AA	Thyroid hormones
E01	Iodine-deficiency-related thyroid disorders and allied conditions		
E02	Subclinical iodine-deficiency hypothyroidism		
E03	Other hypothyroidism		
E05	Thyrotoxicosis [hyperthyroidism]		
E062	Chronic thyroiditis with transient thyrotoxicosis		
E063	Autoimmune thyroiditis		
E065	Other chronic thyroiditis		
E07	Other disorders of thyroid		
E350	Disorders of thyroid gland in diseases classified elsewhere		
E890	Postprocedural hypothyroidism		

**VENOUS AND LYMPHATIC DISEASES**			
ICD-10 codes and labels included in chronic disease category		*ATC-5 codes and labels for drugs associated to chronic disease category. Includes none or any of the following:*	
I780	Hereditary haemorrhagic telangiectasia	C04AD	Purine derivatives
I83	Varicose veins of lower extremities		
I87	Other disorders of veins		
I89	Other noninfective disorders of lymphatic vessels and lymph nodes		

I972	Postmastectomy lymphoedema syndrome
Q820	Hereditary lymphoedema

Only chronic diseases and their associated ATC drug categories resulting in chronic disease or chronic disease-drug categories ≥ 2% prevalence are included in this table. Remaining categories with < 2% prevalence can be sent to readers upon request.

Abbreviations: ACE inhibitors: Angiotensin convertint enzyme inhibitors; COPD: Chronic Obstructive Pulmonary Disease; HMGCoA-reductase : 3-Hidroxi-3-metil-glutaril-CoA reductase.
